# A double-incision endoscopic myotomy of the cricopharyngeal muscle for the management of a large hemi-circumferential Zenker’s diverticulum

**DOI:** 10.1055/a-2307-5809

**Published:** 2024-05-07

**Authors:** Sunil Gupta, Tony He, Jeffrey D. Mosko, Natalia Soledad Causada Calo, Christopher Teshima, Gary R. May

**Affiliations:** 1 10071 Department of Therapeutic Endoscopy, St Michaelʼs Hospital, Toronto, Canada; 2 8539 Department of Gastroenterology and Hepatology, Westmead Hospital, Sydney, Australia

An 81-year-old man with a decade-long history of dysphagia to solids presented to our hospital in extremis. He had lost 20 pounds in weight over 7 months, and more recently had been unable to tolerate clear fluids. A gastroscopy, barium swallow, and computed tomography scan revealed a large hemi-circumferential Zenker’s diverticulum, located 18 cm from the incisors and centered at 9 o’clock ( Fig. 1 **a** ), with minimal passage of contrast into the esophageal lumen observed.

**Fig. 1:**
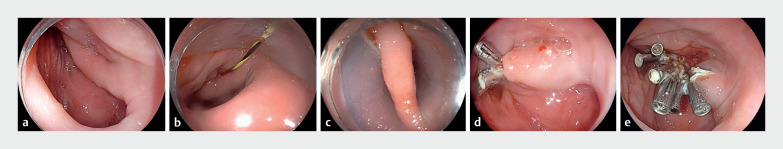
Endoscopic images showing: **a** a large hemi-circumferential Zenker’s diverticulum located 18 cm from the incisors and centered at 9 o’clock; **b** a 0.035-inch guidewire placed to guide a Zenker’s overtube toward the diverticulum; **c** isolation of the muscular septum of the Zenker’s diverticulum; **d** the remnant posterior component of the Zenker’s diverticulum following completion of the first myotomy; **e** complete flattening of the Zenker’s diverticulum following completion of a second myotomy and clip closure.

Owing to a long muscular septum, we proceeded to perform a double-incision endoscopic dissection of the cricopharyngeal muscle ( Video 1 ). Zenker’s diverticulum peroral endoscopic myotomy (zPOEM) was not considered because of the potential need for two tunnels. After the diverticulum had been thoroughly cleaned, a 0.035-inch guidewire was placed into the stomach ( Fig. 1 **b** ). This was used to guide placement of a Zenker’s overtube (ZDO-22-30; Cook Medical, Bloomington, Indiana, USA) into the diverticulum to isolate the muscular septum ( Fig. 1 **c** ). A complete myotomy of the cricopharyngeal muscle was performed with a precut needle-knife (Zimmon knife; Cook Medical). After clip closure, re-evaluation revealed that the posterior component of the diverticulum remained prominent ( Fig. 1 **d** ) and there was still resistance to passage of the endoscope into the esophagus. The Zenker’s overtube was reinserted, isolating the remnant muscular septum. A second complete myotomy of the cricopharyngeal muscle was performed, followed again by clip closure. Subsequently to this, there was flattening of entire diverticulum and easy passage of the gastroscope into the esophagus ( Fig. 1 **e** ). Over the next few days, the patient’s dysphagia to liquids and solids completely resolved.

A double-incision Zenker’s myotomy is performed for the management of a large hemi-circumferential Zenker’s diverticulum.Video 1

Flexible endoscopic dissection of the cricopharyngeal muscle is well established as a safe and effective treatment option for a symptomatic Zenker’s diverticulum 1 . Our novel double-incision myotomy enabled complete flattening of a large hemi-circumferential diverticulum. In our experience, the use of a Zenker’s overtube aided exposure of the remnant septal bridge and facilitated the second myotomy. Further studies pertaining to the clinical outcomes of this approach are required.

Endoscopy_UCTN_Code_TTT_1AO_2AF

## Notes

Endoscopy E-Videos https://eref.thieme.de/e-videos
*E-Videos* is an open access online section of the journal *Endoscopy* , reporting on interesting cases and new techniques in gastroenterological endoscopy. All papers include a high-quality video and are published with a Creative Commons CC-BY license. Endoscopy E-Videos qualify for HINARI discounts and waivers and eligibility is automatically checked during the submission process. We grant 100% waivers to articles whose corresponding authors are based in Group A countries and 50% waivers to those who are based in Group B countries as classified by Research4Life (see: https://www.research4life.org/access/eligibility/ ). This section has its own submission website at https://mc.manuscriptcentral.com/e-videos .

## References

[1] Schoeman S Kobayashi R Marcon N Outpatient flexible endoscopic diverticulotomy for the management of Zenker’s diverticulum: a retrospective analysis of a large single-center cohort Gastrointest Endosc 2023 97 226 231 10.1016/j.gie.2022.09.022 36228698

